# Functional Connectivity Changes in the Insular Subregions of Patients with Obstructive Sleep Apnea after 6 Months of Continuous Positive Airway Pressure Treatment

**DOI:** 10.1155/2023/5598047

**Published:** 2023-02-21

**Authors:** Ting Long, Haijun Li, Yongqiang Shu, Kunyao Li, Wei Xie, Yaping Zeng, Ling Huang, Li Zeng, Xiang Liu, Dechang Peng

**Affiliations:** ^1^Medical Imaging Center, The First Affiliated Hospital of Nanchang University, Jiangxi Province, China; ^2^PET Center, The First Affiliated Hospital of Nanchang University, Jiangxi Province, China

## Abstract

This study was aimed at investigating the functional connectivity (FC) changes between the insular subregions and whole brain in patients with obstructive sleep apnea (OSA) after 6 months of continuous positive airway pressure (CPAP) treatment and at exploring the relationship between resting-state FC changes and cognitive impairment in OSA patients. Data from 15 patients with OSA before and after 6 months of CPAP treatment were included in this study. The FC between the insular subregions and whole brain was compared between baseline and after 6 months of CPAP treatment in OSA. After 6 months of treatment, OSA patients had increased FC from the right ventral anterior insula to the bilateral superior frontal gyrus and bilateral middle frontal gyrus and increased FC from the left posterior insula to the left middle temporal gyrus and left inferior temporal gyrus. Hyperconnectivity was found from the right posterior insula to the right middle temporal gyrus, bilateral precuneus, and bilateral posterior cingulate cortex, which mainly involved the default mode network. There are changes in functional connectivity patterns between the insular subregions and whole brain in OSA patients after 6 months of CPAP treatment. These changes provide a better understanding of the neuroimaging mechanisms underlying the improvement in cognitive function and emotional impairment in OSA patients and can be used as potential biomarkers for clinical CPAP treatment.

## 1. Introduction

Obstructive sleep apnea (OSA) is characterized by recurrent episodes of partial or complete collapse of the upper airway during sleep, resulting in hypoventilation or apnea [1]. Globally, the overall prevalence of OSA ranges between 9% and 38% [2–4]. Daytime sleepiness, sleep fragmentation, intermittent hypoxia, and oxidative stress are associated with varying degrees of cognitive impairment in OSA patients [5]. Thus, substantial studies have been conducted on the underlying neurodegenerative mechanisms of emotional and cognitive impairments in OSA patients. Previous neuroimaging studies have found structural and functional alterations in several brain regions associated with cognitive impairment in OSA patients, including the insula [6–9]. However, the mechanisms underlying the effects of OSA on insular function and cognitive function remain unclear.

The insula is located deep within the lateral sulcus of the Sylvian fissure and is highly interconnected with the frontal, temporal, and parietal brain regions [10]. It performs several functions and serves as a critical convergence point for multiple neural pathway inputs [11–13]. Li et al. showed that the insular cortex is important in OSA development and that activation of the insular cortex increases the control of the nucleus accumbens over the septal nucleus, leading to a reduction in 5-hydroxytryptamine release, ultimately reducing the tone of the chin and tongue muscles, and airway collapse [14]. In a study of OSA structure, it was found that thinning of the insular cortex may be associated with cognitive dysfunction [15]. According to a magnetic resonance spectroscopy study, lower N-acetylaspartate and higher myo-inositol levels in the bilateral insula in OSA patients indicated neuronal damage and enhanced glial cell activation, respectively, explaining the autonomic and cognitive abnormalities in OSA patients [16]. Park et al. studied the insula as seeds using resting-state functional connectivity (FC) and found substantial FC abnormalities in the insula that explained the impairment of autonomic, emotional, and sensorimotor systems in OSA patients [17]. Previous studies have found similar evidence of structural atrophy, dysfunction, and metabolic abnormalities in the insular cortex of OSA patients. However, in the past, the insula has mostly been studied as a whole, ignoring the existence of different functional patterns in the insular subregions.

The insula can be divided into at least three distinct functional regions based on previous cytoarchitectural studies, meta-analyses, and FC studies: the ventral anterior insula (vAI) associated with emotional and chemosensory processing, the dorsal anterior insula (dAI) associated with higher cognitive processing, and the posterior insula (PI) associated with pain, sensorimotor, and language processing [18–20]. Different diseases can lead to altered patterns of intrinsic FC in the insular subregions. Liu et al. discovered three distinct cognitive-related FC patterns in the insular subregions of patients with Alzheimer's disease as compared to healthy subjects, and these distinct resting-state FC patterns were mostly due to impaired FC of different insular subregions to diverse brain networks, such as the default mode network (DMN) [21]. An analysis of FC in the insula of patients with schizophrenia revealed an unorganized pattern, with decreased FC in the dorsal insula associated with cognitive impairment and decreased FC from the vAI to the superior temporal sulcus associated with negative symptoms [22]. Our previous study on OSA found that abnormal FC patterns in the insular subregions were mainly distributed in the brain regions of emotional, cognitive, and sensorimotor functional areas and that abnormal FC was associated with sleep quality [23]. The majority of earlier studies have indicated potential variations in FC patterns in the insular subregions before disease therapy, but little is known about the effects of treatment.

Currently, continuous positive airway pressure (CPAP) is the primary and most effective treatment for OSA [24]. Previous studies have verified the efficacy of CPAP therapy, including improvements in clinical symptoms (daytime sleepiness) and concurrent cognitive dysfunction in OSA patients, and have provided new insights into the neuroimaging mechanisms of brain changes after CPAP treatment [25–27]. After 3 months of CPAP treatment, increased gray matter volume in the hippocampus and frontal lobes of OSA patients was correlated with improvements in memory, attention, and executive function, according to a structure-related study [28]. A diffusion tensor imaging study found almost complete recovery from white matter abnormalities in OSA patients after 12 months of CPAP treatment [29]. Our previous resting-state functional magnetic resonance imaging (rs-fMRI) study found that after 1 month of CPAP treatment, spontaneous brain activity in the bilateral cerebellum posterior lobe, right superior frontal gyrus (SFG), and left precentral gyrus could reverse, and these changes were associated with improvements in cognition and sleepiness, but no structural brain changes were found, suggesting that functional changes may be asynchronous with structural changes [30, 31]. However, no study on the FC of insular subregions after CPAP treatment in OSA patients has been reported.

Based on these aspects, we hypothesized that CPAP treatment could improve FC patterns in different subregions of the insula and that alterations in FC in these insular subregions could be related to cognitive function and emotional processing. To test this hypothesis, we used a spherical seed region of interest- (ROI-) based FC analysis to explore FC changes in the insular subregions of OSA patients before and after CPAP treatment. We then assessed the correlation between FC changes in insular subregions and clinical neurocognitive scales in OSA patients.

## 2. Materials and Methods

### 2.1. Participants

**Figure 1:**
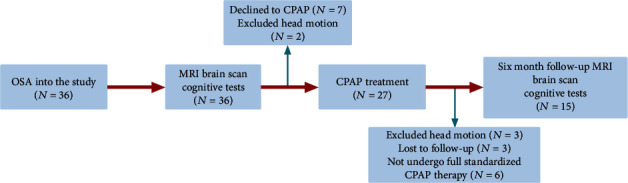
Schematic diagram of OSA patient process in this study.

Before starting CPAP treatment, 36 patients with OSA were recruited and the recruitment process of patients with OSA is shown in Figure 1. Finally, 15 patients with OSA were included in the follow-up analysis. This longitudinal study included 15 subjects in the analysis. Before the study, none of the patients had undergone CPAP. All patients were diagnosed according to the criteria of the American Academy of Sleep Medicine (AASM)'s Clinical Practice Guidelines for Adult OSA published in 2017 [32]. The inclusion criteria were as follows: apnea hypoventilation index (AHI) > 15/h, age between 18 and 60 years, and right-handedness. The exclusion criteria were as follows: (1) sleep disorder diseases other than OSA; (2) history of CPAP treatment; (3) central nervous system, respiratory, and cardiovascular diseases and diabetes mellitus; (4) alcoholism, abuse of prohibited drugs, or use of psychotropic drugs; (5) contraindications (metal implantation in the body and claustrophobia) to MRI examination; and (6) incomplete CPAP treatment and loss to follow-up (16 patients with OSA were excluded). We examined OSA patients using rs-fMRI scans and neuropsychological testing at baseline and 6 months after CPAP treatment.

### 2.2. Polysomnographic Monitoring and Neuropsychological Assessment

Prior to MRI data collection, all subjects underwent polysomnography (PSG) monitoring throughout the night (from 10 p.m. to 6 a.m. the next morning). The day before PSG monitoring, all subjects were instructed to avoid drinking or having coffee. All subjects were monitored at baseline using an Alice 5 LE monitor (Alice 5 LE, Respironics, Orlando, FL, USA). The items recorded included standard electroencephalogram (F3-M2, F4-M1, C3-M2, C4-M1, T3-M2, T4-M1, O1-M2, and O2-M1), chin electromyogram, electrocardiogram, body position, chest and abdominal respiratory movements, and snoring. The PSG parameters evaluated included AHI, mean oxygen saturation (SaO_2_), minimum oxygen saturation (nadir SaO_2_), time with SaO_2_ < 90% as a percentage of total sleep time, sleep latency, sleep efficiency, and total sleep time. According to the AASM guidelines, obstructive apnea is defined as a reduction in airflow of >90% or no airflow for at least 10 s, and hypoventilation is defined as a reduction in airflow of ≥30% during sleep with a 4% or greater drop in arterial oxygen saturation. The AHI refers to the total number of apnea and hypoventilation events that occur in a patient per hour of sleep.

All subjects completed clinical and neuropsychological assessments at baseline and at 6-month follow-up using the Montreal Cognitive Assessment (MoCA), Epworth Sleepiness Scale (ESS), Pittsburgh Sleep Quality Index (PSQI), Hamilton Anxiety Scale (HAMA), and Hamilton Depression Scale (HAMD). The MoCA scale assesses cognitive functioning, including executive functioning, language, attention, calculation, abstraction, naming, memory, and orientation. There is a total score of 30 on the MoCA scale, with a score below 26 indicating impaired cognitive functioning [33]. The ESS is used to assess daytime sleepiness and consists of eight different category scores, each of which is rated from 0 to 3, with a total score of 0–24. The PSQI assesses subjective sleep quality, with a total score of 21, higher scores indicating poorer sleep quality. The HAMA and HAMD scales are used to assess psychiatric symptoms. Total HAMA scores of >29, >21, >7, and <7 indicate possible severe anxiety disorder, definite significant anxiety disorder, possible anxiety disorder, and no anxiety disorder, respectively. Total HAMD scores of >24, >17, and <7 may indicate major depression, mild to moderate depression, and no depressive symptoms, respectively.

### 2.3. Continuous Positive Airway Pressure Treatment

All OSA patients were treated with standard CPAP for at least 6 months at a frequency of at least 4 h per night for at least 5 days per week. OSA patients were treated with a standardized ventilator in an autoadjustment mode (Yuyue 480, Jiangsu, China), which was set to a therapeutic pressure of 4–20 cm H_2_O and automatically programmed for the night of sleep. The ventilator has a built-in subscriber identity module card that automatically stores and records user data, including duration of use, AHI, mask leakage, and blood oxygen, which is analyzed by a professional institution after 6 months.

### 2.4. Magnetic Resonance Data Acquisition

In our hospital, MRI data were collected from all subjects using a German Siemens 3.0T Trio Tim MRI scanner with an 8-channel phased-array head coil. The first acquisition was made the day (7 pm to 9 pm) after PSG monitoring and the subsequent after at least 6 months of qualifying CPAP treatment. Foam pads and ear plugs were used to decrease subject head movement and scanner noise. All subjects must lie on the examination couch with their eyes closed, be awake, and avoid involvement in any specific thought activity prior to scanning. Conventional T1-weighted imaging was first performed using the following parameters: repetition time (TR), 250 ms; echo time (TE), 2.46 ms; thickness, 5 mm; gap 1.5 mm; field of view (FOV), 220 mm × 220 mm; and slices, 19. T2-weighted imaging had the following parameters: TR, 4000 ms; TE, 113 ms; thickness, 5 mm; gap, 1.5 mm; FOV, 220 mm × 220 mm; and slices, 19. The rs-fMRI data were imaged using a gradient-echo planar imaging pulse sequence with the following parameters: TR, 2000 ms; TE, 30 ms; flip angle, 90°; thickness, 4 mm; gap, 1.2 mm; FOV, 230 mm × 230 mm; matrix, 64 × 64; slices, 30; 240 volumes; and scan time, 8 min 6 s. These images were evaluated jointly by two senior radiologists to exclude other brain parenchymal disorders, and no subjects were excluded from the procedure.

### 2.5. Data Preprocessing

This study was based on the MATLAB 2018b (MathWorks, Natick, MA, USA) platform and used the Data Processing and Analysis for Brain Imaging (DPABI, http://rfmri.org/dpabi) to preprocess and analyze rs-fMRI data. The preprocessing steps were as follows: conversion of the image format from DICOM format to NIFTI image format, removal of the first 10 time points of data, slice time correction, head movement correction [34], and subject exclusion if their head motion was >2.5 mm maximum translation or >2.5° maximum rotation (five subjects were excluded). Subsequently, all functional images were spatially normalized to the Montreal Neurological Institute (MNI) using the EPI template and resampled to 3 mm × 3 mm × 3 mm voxel size. Smoothing with a 6 mm full width at half maximum; regression of covariates on brain white matter, cerebrospinal fluid, whole brain mean signal, and head movement parameters; and filtering (0.01–0.08 Hz) were performed.

### 2.6. Defining Seed Points and Functional Connectivity Analysis

According to a previous study on resting-state FC, the entire insula was divided into three distinct functional subregions [35]. Based on several previous studies, six subregions of the bilateral insula were defined and used as spherical seed ROIs, with a 6 mm radius. The MNI coordinates were as follows: left vAI (–33, 13, –7), right vAI (32, 10, –6), left dAI (–38, 6, 2), right dAI (35, 7, 3), left PI (–38, –6, 5), and right PI (35, –11, 6) [36–38]. The time series of each spherical seed was obtained by calculating the average of the rs-fMRI time series of all the voxels in the region. Subsequently, for each seed of each subject, Pearson's correlation coefficients were calculated for the time series with all other voxels in the brain to create a whole-brain FC map for each subject. Finally, to improve the normality of the correlation coefficients, they were converted to *z*-mapping using the Fisher *r*-to-*z* transformation.

### 2.7. Statistical Analyses

The Shapiro–Wilk test was performed to determine the normality of the distribution of demographic and clinical assessment data with SPSS 22.0 software package (SPSS, Inc., Chicago, IL, USA). To examine between-group differences in normally distributed data for pre- and post-CPAP treatment, paired sample *t*-tests were employed, and Wilcoxon signed rank-sum tests were utilized for nonnormally distributed data. Because each clinical parameter was independent of each other, we did not use multiple comparison correction. And *p* < 0.05 was considered statistically significant. For seed-based FC analysis, DPABI software in MATLAB 2018b (MathWorks, Natick, MA, USA) was used. First, a one-sample *t*-test was performed to assess the pattern of distribution of FC in the insular subregions in patients with OSA pre- and post-CPAP. Second, a paired-samples *t*-test with body mass index (BMI) as a covariate was used to compare the differences in the FC of the insular subregions between patients with OSA before and after CPAP treatment. The multiple comparison method uses the Gaussian random field (GRF) theory to correct the results, with a set voxel level of *p* < 0.01 and a cluster level of *p* < 0.05, which was statistically significant. To determine the relationship between the mean FC *z* value of abnormal brain regions and clinical variables, Pearson's correlation or Spearman's correlation analysis was used. And after the Bonferroni correlation, *p* < 0.002 was considered to be statistically significant (6 clinical variables × 4 regions).

## 3. Results

### 3.1. Demographic and Clinical Assessment Information Characteristics

Table 1 summarizes the demographic and clinical characteristics of the pre- and post-CPAP groups. The ESS, MoCA, HAMA, and HAMD scores were significantly lower after 6 months of CPAP treatment than before treatment in OSA patients. However, there was no significant difference in the BMI and PSQI scores between the pre- and post-CPAP OSA groups (*p* > 0.05).

### 3.2. Patterns of Resting-State Functional Connectivity Based on Seed Regions of Interest

**Figure 2:**
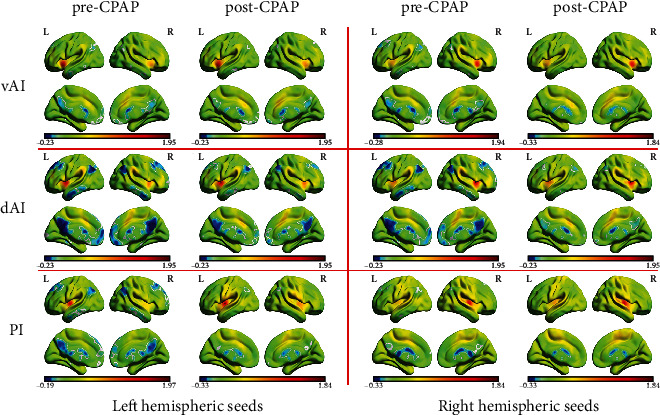
The distribution patterns of resting-state functional connectivity in different insular subregions are highly similar in pre-CPAP OSA and post-CPAP OSA.

Figure 2 shows that patients with OSA pre- and post-CPAP have similar patterns of resting-state FC in different insular subregions.

### 3.3. Difference between Pre- and Post-CPAP OSA Resting-State Functional Connectivity

**Figure 3:**
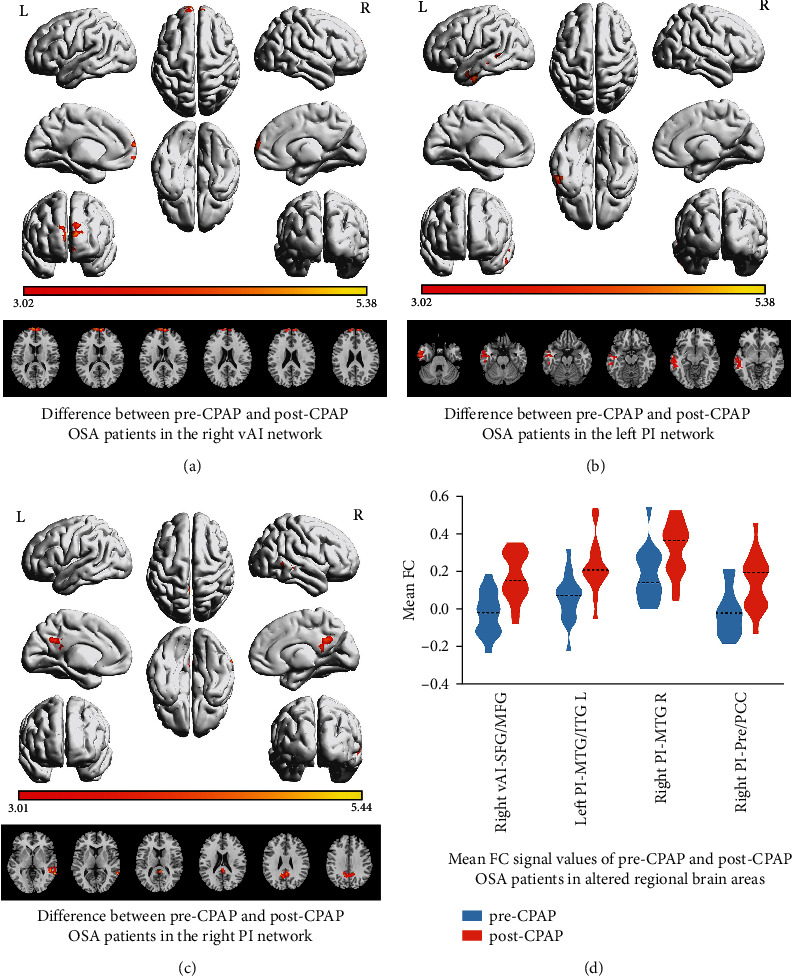
(a) Denotes the FC difference of post-CPAP OSA relative to pre-CPAP OSA based on the right vAI seed. (b) Denotes the FC difference of post-CPAP OSA relative to pre-CPAP OSA based on the left PI seed. (c) Denotes the FC difference of post-CPAP OSA relative to pre-CPAP OSA based on the right PI seed (voxel level *p* < 0.01, cluster-wise *p* < 0.05, two-tailed, GRF corrected). (d) Mean FC *z* signal values of pre- and post-CPAP OSA patients in altered regional brain areas before and after CPAP treatment. Color bars indicate *t*-scores; warm colors indicate areas where the FC value in pre-CPAP OSA is greater than that in post-CPAP. OSA: patients with obstructive sleep apnea; vAI: ventral anterior insula; PI: posterior insula; FC: functional connectivity; SFG: superior frontal gyrus; MFG: middle frontal gyrus; MTG: middle temporal gyrus; ITG: inferior temporal gyrus; Pre: precuneus; PCC: posterior cingulate cortex; L: left; R: right.

The FC between the right vAI and bilateral SFG and medial frontal gyrus (MFG) was considerably higher in the post-CPAP group than in the pre-CPAP group. The FC between the left PI and left middle temporal gyrus (MTG) and left inferior temporal gyrus (ITG) was significantly higher in the post-CPAP group than in the pre-CPAP group. The FC between the right PI and right MTG, bilateral precuneus, and bilateral posterior cingulate cortex (PCC) was also significantly higher in the post-CPAP group than in the pre-CPAP group (Table 2 and Figure 3).

### 3.4. Correlation Analysis

All results of correlation analysis are shown in Supplementary Table 1. And before multiple comparison correction, the connection between FC values and clinical symptoms in the brain regions with significant differences after CPAP treatment is shown in Supplementary Figure 1 (*p* < 0.05). However, there was no significant correlation between FC values and the clinical variables after the Bonferroni correlation with *p* < 0.002, suggesting that the FC changes were largely independent of clinical symptoms alterations.

## 4. Discussion

This study comprehensively investigates the FC changes in the insular subregions of OSA patients after 6 months of CPAP treatment and how these changes relate to neurocognitive and psychosocial functions. Our study showed that compared to pre-CPAP, regions from the right vAI to the bilateral SFG and bilateral MFG, from the left PI to the left MTG and left ITG, and from the right PI to the right MTG, bilateral precuneus, and bilateral PCC exhibited hyperconnectivity, and these regions mainly involved the DMN. In addition, OSA patients showed significant improvement in clinical symptoms after CPAP treatment, including drowsiness symptoms, cognitive function, anxiety, and depressive symptoms. These results suggest that CPAP treatment alters FC patterns in the insular subregions of OSA patients and providing new insights into the neurocognitive mechanisms by which CPAP treatment improves patients with OSA.

The current study found that after CPAP treatment, the FC between the insular subregions and multiple cortices was considerably improved in OSA patients. Anatomical studies have shown that the insula consists of ventral anterior, dorsal anterior, and posterior lobe subregions [39]. Different subregions of the insula have unique functional characteristics based on their different cellular structures and connectivity. The vAI and dAI are primarily involved in endoreceptive awareness, cognition, and emotion processing, whereas the PI is usually involved in processing somatosensory information with emotional or motivational significance [35, 40]. Similar results have been widely confirmed in resting-state studies [41, 42]. In addition, the left and right insula have different functions [43]. Previous studies have confirmed the existence of different FC patterns on the two sides of the insula [44]. These studies anatomically explain the existence of different FC between the insular subregions and different areas of the cerebral cortex. Chen et al. showed that sleep disorders are more likely to lead to functional activation between the anterior insula and salience network [45]. Zhang et al. showed that the disconnection of functional connections between the right anterior insula and medial prefrontal cortex was associated with severity in OSA patients [46]. These results are similar to our previous findings, indicating that factors such as sleep disruption and intermittent hypoxia do not influence the insular subregions in the same way, resulting in different degrees of impaired function in the insular subregions. The study found that after 6 months of CPAP treatment, OSA patients had increased FC between the insular subregions and different brain regions, and we hypothesize that this variability in insular subregion FC may be connected to anatomical features. In addition, the effective improvement of intermittent hypoxia during CPAP treatment may restore FC between the insular subregions and other brain regions to varying degrees.

Our study showed enhanced resting-state FC between the right vAI and bilateral SFG and bilateral MFG after CPAP treatment. The SFG and MFG are critical components of the medial prefrontal cortex (mPFC) and are involved several cognitive and behavioral functions [47, 48]. A previous study reported that neuronal activity between the dorsomedial prefrontal cortex and anterior insula is critical in emotion management techniques [49]. In an animal experiment, chronic hypoxia caused a significant reduction in the number, length, and crossover point of neuronal dendrites in the prefrontal cortex, ultimately causing neuronal remodeling [50]. According to rs-fMRI studies, structural and functional abnormalities in the mPFC of OSA patients are related to cognitive impairment [51]. The findings of this study revealed that after CPAP treatment, the FC between the right vAI and some regions of the mPFC was improved, indicating that the connectivity between the vAI and mPFC was restored. Therefore, we speculate that effective CPAP treatment alters symptoms of intermittent hypoxia, which may reverse neuronal remodeling.

In addition, this study found enhanced FC from the left PI to the left ITG and left MTG and from the right PI to the right MTG. The temporal cortex is involved in various processes such as memory, language, and emotion processing [52–54]. Previous studies have found lower mean diffusivity values in the temporal lobe display area in OSA patients than in healthy controls, and the pathological mechanisms of this injury may include ischemic and hypoxia-induced processes [55]. A decrease in gray matter volume in the bilateral temporal cortex areas was identified in structurally relevant studies in OSA patients [56–58]. Another study found that after 6 weeks of CPAP treatment, mean diffusivity values in the temporal lobe were lowered and cerebral blood flow increased, suggesting that CPAP treatment can promote the repair and recovery of brain injury in OSA patients [59]. Similar to previous findings, we hypothesized that enhanced FC between brain regions may reflect the activation of repair mechanisms and enhanced neural compensatory responses [60].

The precuneus is extensively involved in higher-order cognitive functions and in the regulation of negative emotions [61, 62]. Anatomically, the PCC is a highly connected and metabolically active area that is involved in visuospatial processing and episodic memory [63–65]. In a positron emission tomography study, increased A*β* amyloid deposition in the precuneus and PCC regions was found to predispose most patients with severe OSA to early cognitive impairment [9]. Previous studies have found that glucose metabolism in the precuneus and PCC is lower in patients with sleep disorders and that this low metabolic pattern is associated with sleep arousal [66]. The enhanced FC between the PI, precuneus, and PCC found in this study may be associated with improved cognitive function, although we did not find changes in FC associated with cognitive scales.

In addition, brain regions, such as the mPFC, MTG, PCC, and precuneus, are important components of the DMN [67]. The DMN is characterized by the fact that it is preferentially active when the individual is not focused on the external environment and is involved in a broad range of higher cognitive activities [68]. The DMN can be divided into two functionally distinct subnetworks: a pre-subnetwork centered on the mPFC and a post-subnetwork centered on the PCC [69]. In OSA patients, reduced FC in the pre-DMN and compensatory higher FC in the post-DMN have been reported [51]. A previous study has found that OSA patients exhibit abnormalities in FC between several DMN subregions and abnormalities in spontaneous activity and that these FC changes are related to the severity of OSA and degree of hypoxia [70–73]. A longitudinal study found that CPAP could effectively improve cerebral function by increasing DMN connectivity [74]. Similar to these results, we found enhanced FC between the insular subregions and multiple DMN subregions, and we hypothesize that functional activation between the insular subregions and DMN may be associated with improvements in cognitive function and mood disorders; however, the exact mechanism underlying this needs further investigation.

## 5. Limitations

This study has some limitations. First, this study only evaluated the treatment effect of CPAP after 6 months, and further studies assessing long-term treatment effects are required. Second, relatively few patients were treated with CPAP, and future large-sample multicenter studies should be conducted. Finally, the majority of our patients were moderate-to-severe male OSA patients, which limits their general applicability to the whole population.

## 6. Conclusions

We observed FC changes between the insular subregions and whole brain in patients with moderate-to-severe OSA after 6 months of CPAP treatment, and these FC changes mainly involved the DMN, providing new insights into the possible neuroimaging mechanisms underlying improved cognitive impairment and mood regulation in OSA patients.

## Figures and Tables

**Table 1:** Population and clinical characteristics of participants.

Characteristic	Pre-CPAP (*N* = 15)	Post-CPAP (*N* = 15)	*p* value
Gender (male/female)	14/1	14/1	/
Age (years)	41.53 ± 7.39	/	/
Education (years)	13.4 ± 3.04	/	/
AHI (hour)	47.71 ± 18.26	2.8 (1.5, 5)	<0.001^a^
BMI (kg/m^2^)	26.82 ± 3.36	26.66 ± 3.13	0.664^b^
Nadir SO_2_ (%)	72.0 ± 10.24	/	/
Mean SO_2_ (%)	94.73 ± 2.45	/	/
SaO_2_ < 90%	11.93 ± 10.47	/	/
Sleep efficiency (%)	81.27 ± 8.65	/	/
ESS (scores)	10.0 ± 5.46	6.4 ± 2.82	0.028^b^
HAMA (scores)	7 (5,8)	2.67 ± 2.16	0.001^a^
HAMD (scores)	4.8 ± 2.48	2.53 ± 1.36	0.013^b^
PSQI (scores)	6.07 ± 1.91	5 (4, 10)	0.451^a^
MoCA (scores)	25 (20, 26)	25.4 ± 3.18	0.012^a^
MoCA: visual space and execution	4 (3, 5)	5 (4, 5)	0.363^a^
MoCA: naming	3 (3, 3)	3 (3, 3)	>0.999^a^
MoCA: delayed memory	1.73 ± 1.22	2.53 ± 1.51	0.005^b^
MoCA: attentional function	6 (5, 6)	6 (5, 6)	>0.999^a^
MoCA: language	3 (2, 3)	2 (2, 3)	>0.999^a^
MoCA: abstract	2 (1, 2)	2 (1, 2)	0.625^a^
MoCA: orienteering	6 (6, 6)	6 (6, 6)	0.750^a^

Data are presented as mean ± SD or median (P25, P75). ^a^Wilcoxon's signed-rank test. ^b^Paired samples *t*-test. HC: healthy controls; pre-CPAP OSA: OSA patients before CPAP treatment; post-CPAP OSA: OSA patients after CPAP treatment; BMI: body mass index; AHI: apnea hypopnea index; SaO_2_: oxygen saturation; SaO_2_ < 90%: percentage of total sleep time spent at oxygen saturation < 90%; ESS: Epworth Sleepiness Scale; HAMA: Hamilton Anxiety Scale; HAMD: Hamilton Depression Scale; PSQI: Pittsburgh Sleep Quality Index; MoCA: Montreal Cognitive Assessment.

**Table 2:** Brain areas showing functional connectivity differences with insular subregions between pre-CPAP and post-CPAP OSA.

Seed-ROIs	Brain areas	*L*/*R*	MNI coordinates			Voxel	*t* value
			*X*	*Y*	*Z*		
Right vAI	SFG/MFG	L/R	-6	66	-6	246	5.3826
Left PI	MTG/ITG	L	-57	3	-30	477	5.9342
Right PI	MTG	R	63	-48	3	144	5.4395
	Pre/PCC	L/R	3	-42	18	262	4.9679

Voxel-level (*p* < 0.01), cluster-wise (*p* < 0.05), two-tailed, and GRF-corrected. Pre-CPAP: OSA patients before CPAP treatment; post-CPAP: OSA patients after CPAP treatment; vAI: ventral anterior insula; PI: posterior insula; SFG: superior frontal gyrus; MTG: middle temporal gyrus; ITG: inferior temporal gyrus; Pre: precuneus; PCC: posterior cingulate cortex; L: left; R: right.

## Data Availability

The datasets presented in this article are not readily available because these data are related to the follow-up longitudinal study of our research group, and the datasets are still being replenished. Requests to access the datasets should be directed to TL (longting199812@163.com).

## Supplementary Materials

Supplementary Materials Supplementary Table S1: correlations between insular functional connectivity and clinical characteristic in post-CPAP OSA patients. Supplementary Figure S1: the significant correlation with *P* < 0.05 before multiple comparison correction between insular functional connectivity and clinical assessments in OSA patients after CPAP.
