# Supplementary material for: Functional Connectivity Changes in the Insular Subregions of Patients with Obstructive Sleep Apnea after 6 Months of Continuous Positive Airway Pressure Treatment
Source: Neural Plast. 2023 Feb 21;2023:5598047. doi: 10.1155/2023/5598047 (PMC9974286; doi:10.1155/2023/5598047)
Supplement: Supplementary Materials — Supplementary Table S1: correlations between insular functional connectivity and clinical characteristic in post-CPAP OSA patients. Supplementary Figure S1: the significant correlation with P < 0.05 before multiple comparison correction between insular functional connectivity and clinical assessments in OSA patients after CPAP. [file 5598047.f1.docx]

**Supplementary Material**

**Supplementary Table S1：**Correlations between insular functional connectivity and clinical characteristic in post-CPAP OSA patients.

| FC between brain regions | Correlation | ESS | HAMA | HAMD | MoCA | PSQI | BMI |
| --- | --- | --- | --- | --- | --- | --- | --- |
| Right vAI-bilateral superior frontal gyrus/medial frontal gyrus | r-values | 0.248 | -0.181 | -0.086 | -0.259 | -0.034 | 0.407 |
|  | P-values | 0.373 | 0.518 | 0.760 | 0.351 | 0.903 | 0.132 |
| \| Left PI-left middle temporal gyrus/inferior temporal gyrus \| \| --- \| \|  \| | r-values | 0.039 | 0.110 | -0.164 | -0.045 | -0.304 | 0.248 |
|  | P-values | 0.891 | 0.696 | 0.558 | 0.872 | 0.271 | 0.373 |
| Right PI-right middle temporal gyrus | r-values | 0.027 | -0.019 | -0.293 | 0.218 | 0.125 | -0.071 |
|  | P-values | 0.924 | 0.946 | 0.290 | 0.435 | 0.658 | 0.802 |
| Right PI-bilateral precuneus/posterior cingulate cortex | r-values | 0.096 | -0.573 | -0.611 | -0.246 | -0.203 | -0.152 |
|  | P-values | 0.733 | **0.026^*^** | **0.016^*^** | 0.378 | 0.469 | 0.588 |

Note: ^*^*P* < 0.05 was statistically significant. post-CPAP OSA, OSA patients after CPAP treatment; vAI, ventral anterior insula; PI, posterior insula; FC, functional connectivity; BMI, body mass index; ESS, Epworth Sleepiness Scale; HAMA, Hamilton Anxiety Scale; HAMD, Hamilton Depression Scale; PSQI, Pittsburgh Sleep Quality Index; MoCA, Montreal Cognitive Assessment.


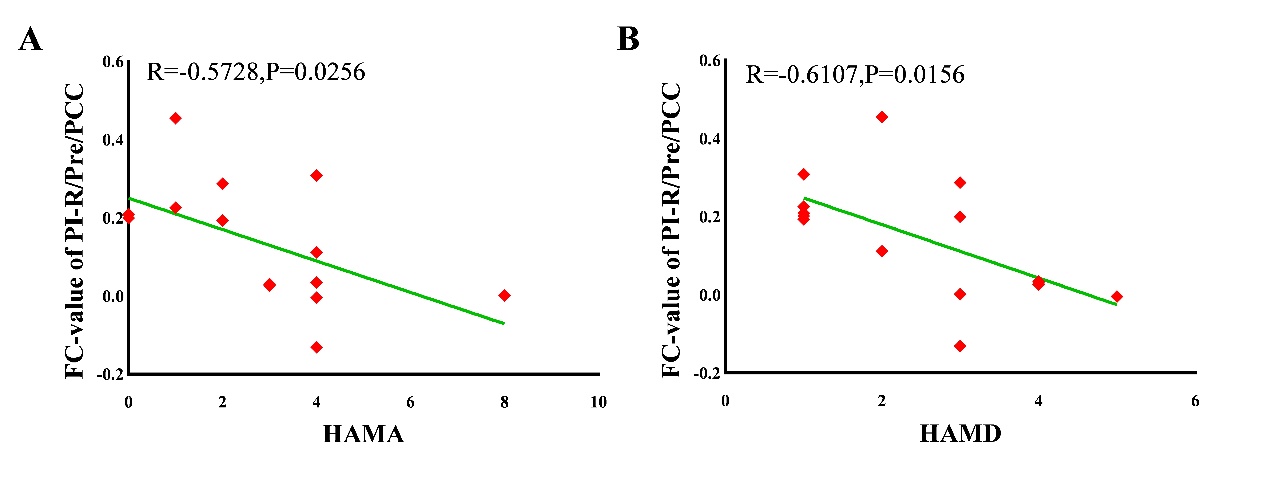


**Supplementary** **Figure S1**|The significant correlation with *P* < 0.05 before multiple comparison correction between insular functional connectivity and clinical assessments in OSA patients after CPAP. FC, functional connectivity; PI, posterior insula; Pre, precuneus; PCC, posterior cingulate cortex; HAMA, Hamilton Anxiety Scale; HAMD, Hamilton Depression Scale; R, right.
